# Decreased expression of CD200R3 on mouse basophils as a novel marker for IgG1-mediated anaphylaxis

**DOI:** 10.1002/iid3.67

**Published:** 2015-06-16

**Authors:** Hiroshi Iwamoto, Takeshi Matsubara, Yuki Nakazato, Kazuyoshi Namba, Yasuhiro Takeda

**Affiliations:** Nutritional Science Institute, Morinaga Milk Industry Co., Ltd.Zama, Kanagawa, 252-8583, Japan

**Keywords:** basophil activation test, CD200R1, CD200R3, IgG-mediated anaphylaxis, mouse basophil

## Abstract

IgE-mediated mast cell activation is the trigger of anaphylaxis in humans, whereas it is known that not only IgE but also IgG can induce anaphylaxis in mice. In our preliminary experiments, the expression of a murine basophil identification marker, CD200R3, on antigen-sensitized basophils decreased following specific antigen challenge. Interestingly, this decrease did not always correspond with increased expression of the IgE-mediated basophil activation marker CD200R1. Since IgG as well as IgE plays a role in mouse anaphylaxis, we hypothesized that the observed decrease in CD200R3 on basophils was caused by IgG-mediated cell activation. We attempted to establish whether CD200R3 is a marker of IgG-mediated basophil activation and if its expression is correlated with anaphylaxis in a mouse model. Mouse basophils were stimulated via Fc∊Rs and/or FcγRs, and levels of CD200R1 and CD200R3 were analyzed by flow cytometry. Basophils derived from naive mice were challenged with a natural antigen, β-lactoglobulin, after passive sensitization with anti-β-LG serum or IgG/IgG subclass-depleted antiserum. Systemic anaphylaxis was induced by i.v. injection of anti-FcγRIII/II monoclonal antibody, and CD200R3 expression on peripheral basophils was assessed. Stimulation via Fc∊Rs induced a significant increase in CD200R1 expression but had only a small effect on that of CD200R3. However, anti-FcγRIII/II stimulation reduced CD200R3 expression markedly. In passive sensitization experiments, down-regulation of CD200R3 induced by antigen challenge was strongly negated by the depletion of IgG or IgG1 from antiserum. Intravenous injection of anti-FcγRIII/II induced CD200R3 down-regulation on peripheral basophils, together with a drop in rectal temperature. Lowered CD200R3 expression on basophils is induced by IgG-mediated stimulation via FcγRs. Use of CD200R1 and CD200R3 as activation markers enables the evaluation of murine basophil activation mediated by IgE and IgG, respectively.

## Introduction

Allergic diseases have become an increasingly challenging public health problem worldwide, particularly in developed countries. The World Allergy Organization's White Book on Allergy, for example, points out that more than 20% of the population of most developed countries suffers from some form of allergic disease 1. Of all allergic manifestations, anaphylaxis is the most serious and potentially life-threatening clinical condition. Causative allergens such as drugs or those derived from food, bind to antigen-specific IgE on mast cells, and cross-link high-affinity IgE receptors (Fc∊RIs), leading to activation and degranulation of the cell. Chemical mediators released from mast cells, such as histamine, cause vascular hyperpermeability, hypothermia, and a drop in blood pressure, and lead to systemic anaphylaxis.

IgE-mediated mast cell activation triggers anaphylaxis in human subjects, whereas it is known that not only IgE (the classical pathway), but also IgG (the alternative pathway) can induce anaphylaxis in mouse models 2–6. Although it has been suggested that basophils 5–8, macrophages 7,9, and neutrophils 7,10 function as effector cells in murine IgG-mediated systemic anaphylaxis, their reported roles vary depending on the investigator and experimental model employed. In order to clarify the importance of these cells in anaphylaxis, it would be useful to examine their respective IgG-mediated activation using specific markers. On this subject, Khodoun 11 reported that the expression of Fcγ receptor III (FcγRIII) on mouse neutrophils decreases during IgG-mediated but not IgE-mediated anaphylaxis, suggesting that FcγRIII can be used as an IgG-mediated neutrophil activation marker. However, no activation markers specific to macrophages or basophils have been identified.

Basophils are uncommon leucocytes, making up less than 1% of peripheral blood cells. Since basophils express Fc∊RI and release histamine in response to its cross-linkage, many researchers have regarded these leucocytes as substitutes for tissue mast cells. However, recent findings that basophils generate a large amount of Th2 cytokines such as IL-4 and IL-13 12–14 have made these cells important players in allergic inflammation. Basophils generate IL-4 and IL-13 following IgE-mediated activation, and these cytokines promote eosinophilic infiltration in surrounding tissue and induce chronic inflammation 8,15.

In clinical practice, the basophil activation test (BAT) has been noted for its usefulness in diagnosing IgE-mediated allergic diseases 16. BAT is an in vitro test of a patient's peripheral basophils, which express Fc∊RI on their surface and are sensitized with antigen-specific IgE. Allergen-induced basophil activation is estimated by measuring the up-regulation of CD63 or CD203c by flow cytometry 17–19. In particular, BAT using CD203c as an activation marker is reported to show a high correlation with clinical state in patients with food 20,21 and bee venom 22 allergies.

In addition, it has been reported that mouse basophils increase the expression of CD200R1 23 and CD41 24 as a result of IgE-mediated activation. Therefore, we concluded that the BAT system would be helpful in the evaluation of allergic reactions in mice. In the process of establishing a murine BAT system using CD200R1 as an activation marker, we found that mouse basophils decrease the expression of their identifying marker, CD200R3, upon activation. When basophils derived from immunized mice were activated by antigens, a decrease in CD200R3 was observed more often than an increase in CD200R1. Immunization of an antigen with an adjuvant (aluminum hydroxide, alum) can induce the production of antigen-specific IgE antibodies; however, the IgG isotype predominates 25. IgG induces anaphylaxis in mice 2–6, as does IgE. Therefore, we hypothesized that the decrease in CD200R3 expression observed on basophils is caused by IgG-mediated cell activation. In the present study, we show that CD200R3 is the marker of IgG1-mediated basophil activation and that its behavior is independent of CD200R1 expression.

## Materials and Methods

### Antigen

Bovine β-lactoglobulin (β-LG) was purified from fresh cow's milk according to the method of Yoshida 26.

### Animals

Three-, eight-, or ten-week-old female BALB/c specific pathogen-free (SPF) grade mice were obtained from CLEA Japan (Tokyo, Japan) and maintained under SPF conditions. All mice were acclimatized for at least 1 week and had ad libitum access to water and cow's milk-free commercial chow (Labo MR Stock; Nihon Nosan Kogyo, Kanagawa, Japan). All animal experiments were approved by the Institutional Animal Care and Use Committee of Morinaga Milk Industry Co., Ltd.

### Immunization of mice and antiserum preparation

Four-week-old mice were immunized twice or three times at 2-week intervals by i.p. injection of 10 µg β-LG with 4 mg of alum. These immunized mice were used as blood donors in the basophilic activation procedure (see below) and as the source of antiserum. To prepare antiserum, postcaval blood was collected using a syringe under general anesthesia with pentobarbital (Somnopentyl; Kyoritsu Seiyaku, Tokyo, Japan), 1 week after the second immunization.

### Basophilic activation and flow cytometry

Heparinized whole blood samples were collected from the tail veins of naive or immunized mice between the ages of 11 and 22 weeks. For the naive mice, pooled blood aliquots (50 µL) were pre-incubated at 37°C for 15 min and subsequently incubated with 50 µL of media, phosphate-buffered saline containing 1% horse serum (PBS-HS), mixed with either goat anti-mouse IgE (Bethyl Laboratories, Montgomery, TX, USA), anti-mouse FcγRIII/II monoclonal antibody (mAb; clone 2.4G2, rat IgG2b, κ; BD Biosciences, San Jose, CA, USA), or isotype control (clone A95-1, rat IgG2b, κ; BD Biosciences). Aliquots of blood from immunized mice were incubated with β-LG in the same manner. After the incubation period, blood samples were mixed with 10 µL of 20 mM EDTA and placed on ice for 10 min, followed by centrifugation (500×*g* for 5 min at 4°C).

The precipitated cells were blocked with 15% HS in PBS for 30 min on ice, and then stained with APC-conjugated anti-mouse IgE (Columbia Biosciences, Columbia, MD, USA), PerCP/Cy5.5-conjugated anti-mouse CD49b, PE-conjugated anti-mouse CD200R1 (both from BioLegend, San Diego, CA, USA), and FITC-conjugated anti-mouse CD200R3 (Hycult Biotech, Uden, Netherlands) for 30 min on ice. The cells were subjected to ammonium-chloride-potassium buffer (150 mM NH_4_Cl, 10 mM KHCO_3_, 10 µM EDTA) to lyse erythrocytes, and washed three times with PBS-HS. The cells were re-suspended in PBS-HS and analyzed using a FACSCanto II flow cytometer with FACSDiva software (both from BD Biosciences). Relative expression levels were calculated from mean fluorescence intensities (MFIs).

### Passive sensitization of whole blood followed by antigen challenge

Heparinized whole blood samples were collected from naive mice as described above. Mouse anti-β-LG serum was serially diluted in PBS-HS and added to 50 µL of blood. After incubating at 37°C for 2 h, passively sensitized blood samples were mixed with equal amounts of β-LG (1 µg/mL), followed by further incubation.

### Depletion of IgG and IgG-subclasses from antiserum

Mouse anti-β-LG serum was diluted ten-fold in PBS-HS. For the depletion of IgG, diluted antiserum samples were then mixed with an equal amount of Protein G Sepharose 4 Fast Flow (GE Healthcare, Uppsala, Sweden) or Sepharose 4B (SIGMA-ALDRICH, Saint Louis, MO, USA), and incubated for 2 h at room temperature on a rotating platform. Following incubation, the antiserum samples were recovered by centrifugation (500×*g* for 5 min at room temperature). In order to deplete IgG-subclasses, Streptavidin Sepharose High Performance (GE Healthcare) was mixed with double its volume of each of the following biotinylated rat mAbs at a concentration of 0.5 mg/mL. IgG-subclass specific antibodies (BD Biosciences); anti-mouse IgG1 (clone A85-1, IgG1, κ), anti-mouse IgG2a (clone R19-15, IgG1, κ), anti-mouse IgG2b (clone R12-3, IgG2a, κ), and anti-mouse IgG3 (clone R40-82, IgG2a, κ), and isotype controls (BioLegend) for IgG1 (clone RTK2071) and IgG2a (clone RTK2758). The antibody-bound beads were subsequently washed five times with PBS-HS. Incubation with mouse anti-β-LG serum followed by recovery of the serum samples was carried out as described above. The depletion of IgG and IgG-subclasses from the antiserum was confirmed by ELISA.

### Induction and evaluation of systemic anaphylaxis

Mice were injected intravenously with 100 µg of 2.4G2 or isotype control A95-1 in 200 µL PBS (both reagents were azide-free and low endotoxin-grade; BD Biosciences). Anaphylaxis was evaluated by the measurement of rectal temperature using a digital thermometer (TD-300; Shibaura Electronics, Saitama, Japan).

### Statistical analysis

Data are expressed as means ± SDs and were analyzed using a two-tailed paired Student's *t*-test. A *P*-value lower than 0.05 was considered significant.

## Results

### Changes in expression levels of CD200R1 and CD200R3 are induced by basophilic activation

Previous studies have reported that the expression of CD200R1 on mouse basophils increases in response to antigen-specific and anti-IgE stimulation in a mouse allergy model 23, and Ba103, specific to CD200R3, has been established as a basophil-recognizing mAb 27,28. Based on these reports, we attempted to establish a BAT system for a mouse model of milk allergy. Whole blood from β-LG-immunized mice was incubated with the corresponding antigen (β-LG) and basophil activation was evaluated by flow cytometry using CD200R3 as one of the identification markers and CD200R1 as a marker of activation.

**Figure 1:**
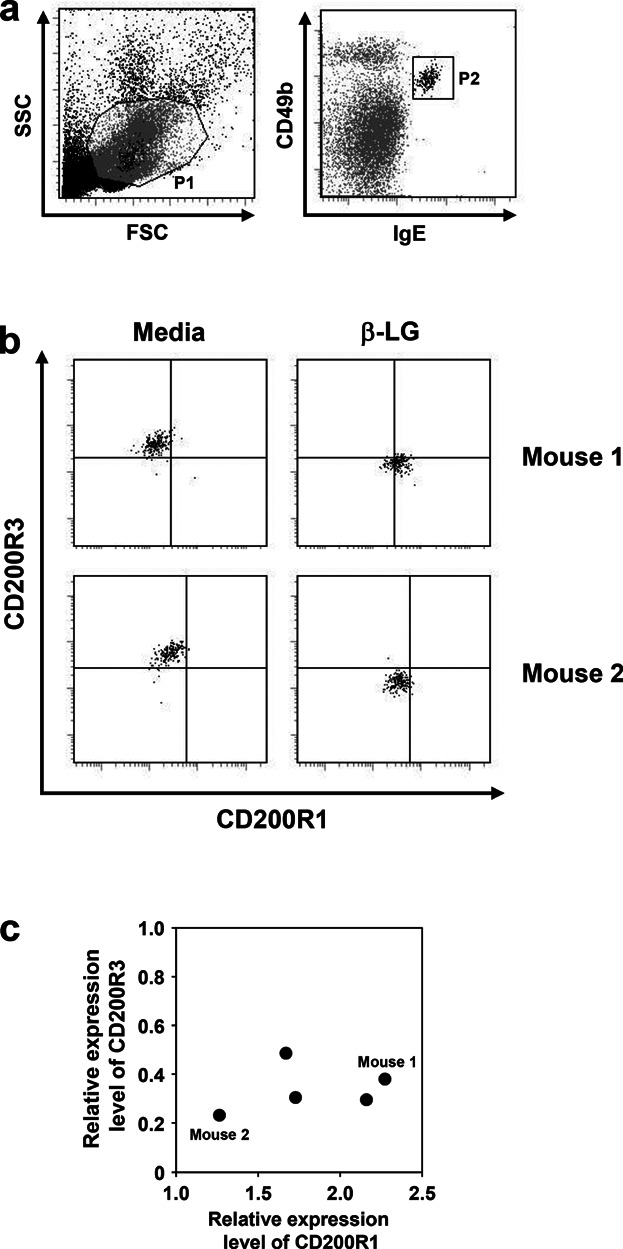
Changes in expression levels of CD200R1 and CD200R3 on blood basophils of β-LG-immunized mice following antigen stimulation. Five BALB/c mice were immunized with β-LG. Peripheral blood was incubated with or without β-LG at 37°C for 2 h. The cells were stained as described in Materials and Methods. (a) Gating strategy for identification of basophils by flow cytometry. Peripheral white blood cells were initially gated based on forward scatter and side scatter (region P1). Region P1 was further gated based on CD49b and IgE (region P2), and the cells in region P2 were deemed to be basophils. (b) Representative dot plots showing the expression of CD200R1 and CD200R3 on basophils following stimulation with (right) or without (left) β-LG. (c) Scatter plots graph showing the relative expression levels of CD200R1 and CD200R3 following stimulation with β-LG. All of the experimental data (five mice) are shown.

When CD49b and CD200R3 were used as identification markers, basophils stimulated with β-LG were somewhat easily confused with other cells (data not shown). Therefore, we used CD49b and IgE to identify basophils (Fig. 1a), and re-assessed the expression levels of CD200R1 and CD200R3 on these cells. This combination of markers facilitated detection of basophils under antigen-stimulated conditions, during which the up-regulation and down-regulation of CD200R1 and CD200R3, respectively, were observed (Fig. 1b). The up-regulation of CD200R1 did not always correspond to the down-regulation of CD200R3, that is, whereas the down-regulation of CD200R3 was clearly observed in all mice, the degree of up-regulation of CD200R1 was varied depending on individual mice (Fig. 1c). These findings suggest that different mechanisms regulate the expression of CD200R1 and CD200R3 under antigen-stimulation.

### Basophil surface expression of CD200R3 decreases upon IgG receptor-mediated cellular activation

**Figure 2:**
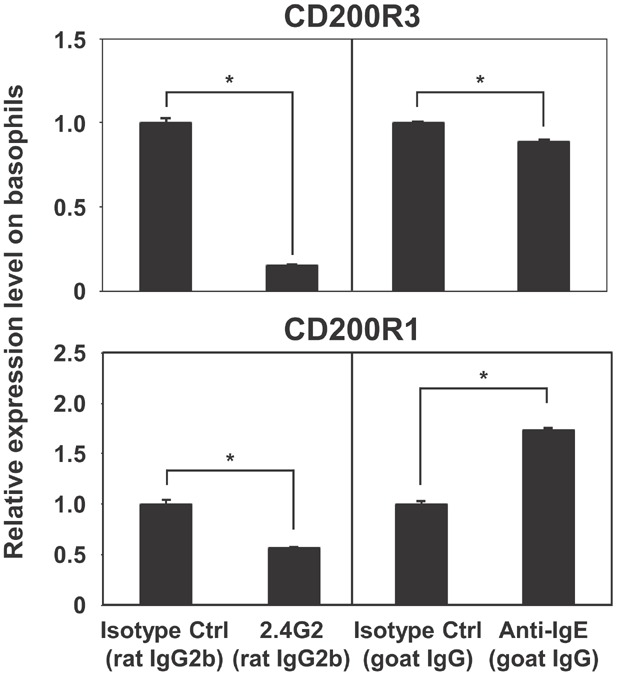
Increased expression of CD200R1 and decreased expression of CD200R3 on basophils in response to IgE and IgG receptor-mediated activation, respectively. Peripheral blood was incubated at 37°C for 2 h with 100 ng/mL anti-mouse FcγRIII/II (2.4G2), 300 ng/mL goat anti-mouse IgE, or the respective isotype control antibody. Relative expression levels of CD200R1 and CD200R3 on basophils are shown. Data are represented as means ± SDs (*n* = 3). Asterisks indicate significant differences (*P *< 0.01).

Mouse basophils are activated by both IgE- and IgG-mediated systems 5,6. Although immunization of an antigen with an adjuvant (alum) can induce the production of antigen-specific IgE antibodies, IgG antibodies predominate 25. Therefore, we hypothesized that the down-regulation of CD200R3 on basophils was induced by IgG-mediated stimulation. To address this hypothesis, we evaluated the change in basophilic surface expression levels of CD200R1 and CD200R3 following IgE- and IgG-mediated stimulation. Treatment of whole blood samples from naive mice with an anti-IgE antibody resulted in the up-regulation of CD200R1, whereas only a small change in the expression of CD200R3 was observed (Fig. 2). The expression level of CD200R3 on basophils was markedly reduced by stimulation with anti-FcγRIII/II mAb (Fig. 2). This antibody also induced down-regulation of CD200R1, albeit to a lesser extent than CD200R3.

### Decreased expression of CD200R3 on basophils is dose- and time-dependent

**Figure 3:**
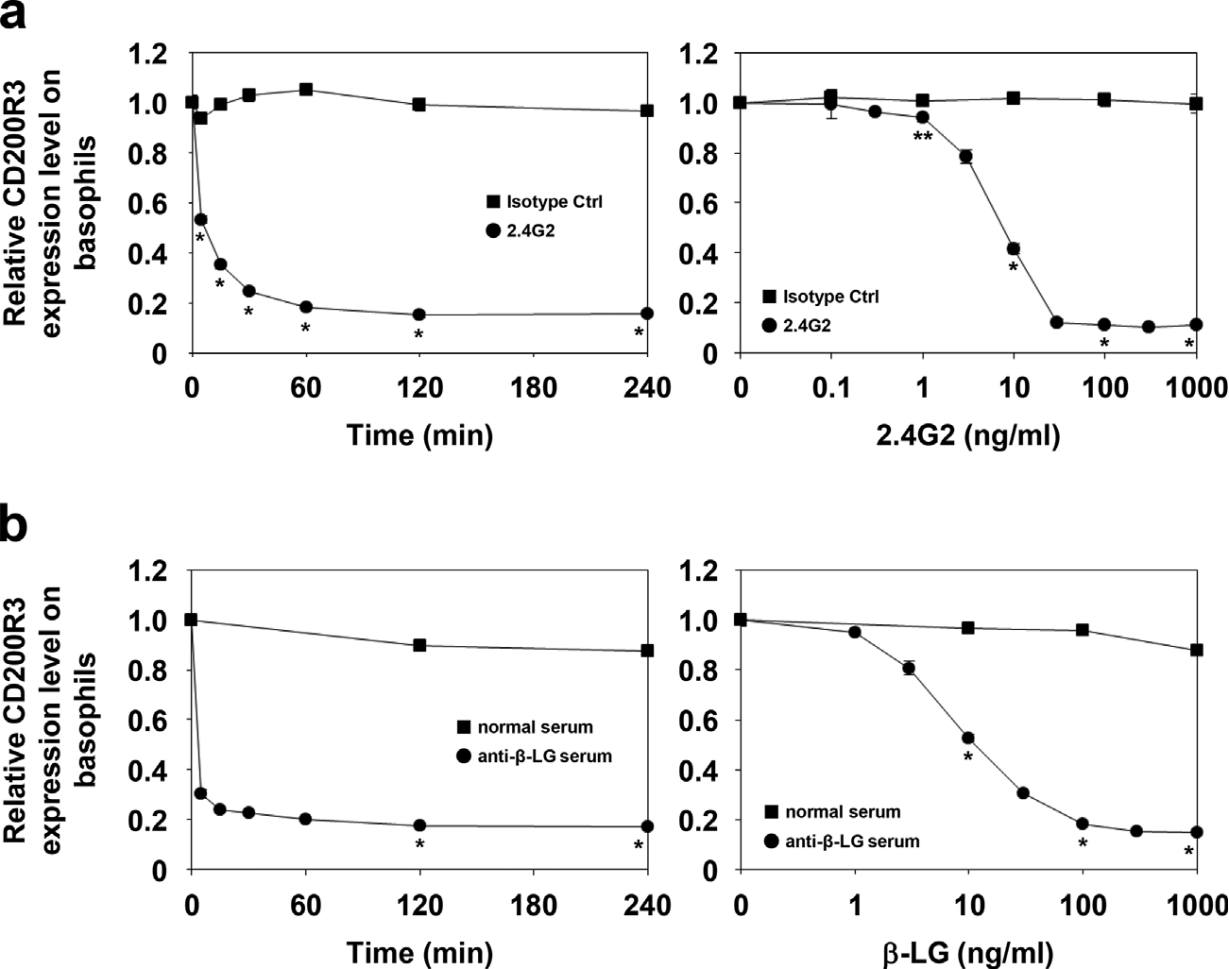
Reduced surface expression of CD200R3 on basophils in a dose- and time-dependent manner after stimulation with anti-mouse FcγRIII/II (2.4G2) or β-LG following passive sensitization with the corresponding antiserum. (a) Peripheral blood was incubated with 100 ng/mL 2.4G2 or isotype control (left panel), as well as various concentrations of antibodies for 2 h (right panel). (b) Peripheral blood was passively sensitized with mouse anti-β-LG serum or normal serum, corresponding to one-twentieth of the blood sample volume. Following sensitization, samples were incubated with 1 μg/mL β-LG (left panel). Passively sensitized samples were also incubated with various quantities of β-LG for 2 h (right panel). The expression of CD200R3 on basophils after stimulation was analyzed by flow cytometry and is shown as relative expression levels. Data are represented as means ± SDs (*n* = 3). Asterisks indicate significant differences between the two groups at each time point (left panels), or those compared to media alone (right panels; *P < 0.01, **P < 0.05).

We next evaluated whether the decreased basophilic surface expression of CD200R3 was dose- and time-dependent. Stimulation of whole blood samples with anti-FcγRIII/II mAb resulted in a marked decrease in the expression of CD200R3 on basophils, and this reduced level was maintained for 4 h (Fig. 3a, left panel). This reduction was dependent on antibody concentration (Fig. 3a, right panel).

When whole blood samples were passively sensitized with mouse anti-β-LG serum and subsequently challenged with β-LG, the level of CD200R3 decreased in a time-dependent manner, in a pattern similar to that of FcγRIII/II stimulation (Fig. 3b, left panel). The degree to which CD200R3 expression decreased corresponded to the dose of the antigen (Fig. 3b, right panel).

### IgG- or IgG1-specific depletion influences basophilic surface expression of CD200R3

**Figure 4:**
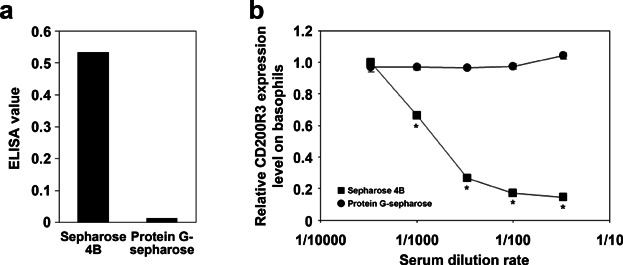
Basophil CD200R3 expression levels following IgG-specific depletion from antiserum used for passive sensitization. (a) Mouse antiserum was treated with either Protein G Sepharose 4FF or Sepharose 4B and β-LG-specific IgG were measured by ELISA. (b) Mouse peripheral blood was sensitized with antiserum treated with either Protein G Sepharose 4FF or Sepharose 4B, followed by antigen challenge with 1 μg/mL β-LG for 2 h. The expression level of CD200R3 was analyzed by flow cytometry and is given as relative expression level. Data are represented as means ± SDs (*n* = 3). Significant differences between groups are indicated by asterisks (*P < 0.01).

To determine whether the alteration in basophilic surface expression levels of CD200R3 was IgG-specific, BAT was performed after passive sensitization with IgG-depleted anti-β-LG serum. The depletion of IgG antibody from antiserum was successful by Protein G Sepharose treatment (Fig. 4a). The expression levels of CD200R3 on basophils sensitized with anti-β-LG serum decreased following antigen challenge in an antiserum concentration-dependent manner. However, after depletion of IgG from the antiserum using Protein G Sepharose, no alteration in the level of CD200R3 was observed after antigen challenge (Fig. 4b).

**Figure 5:**
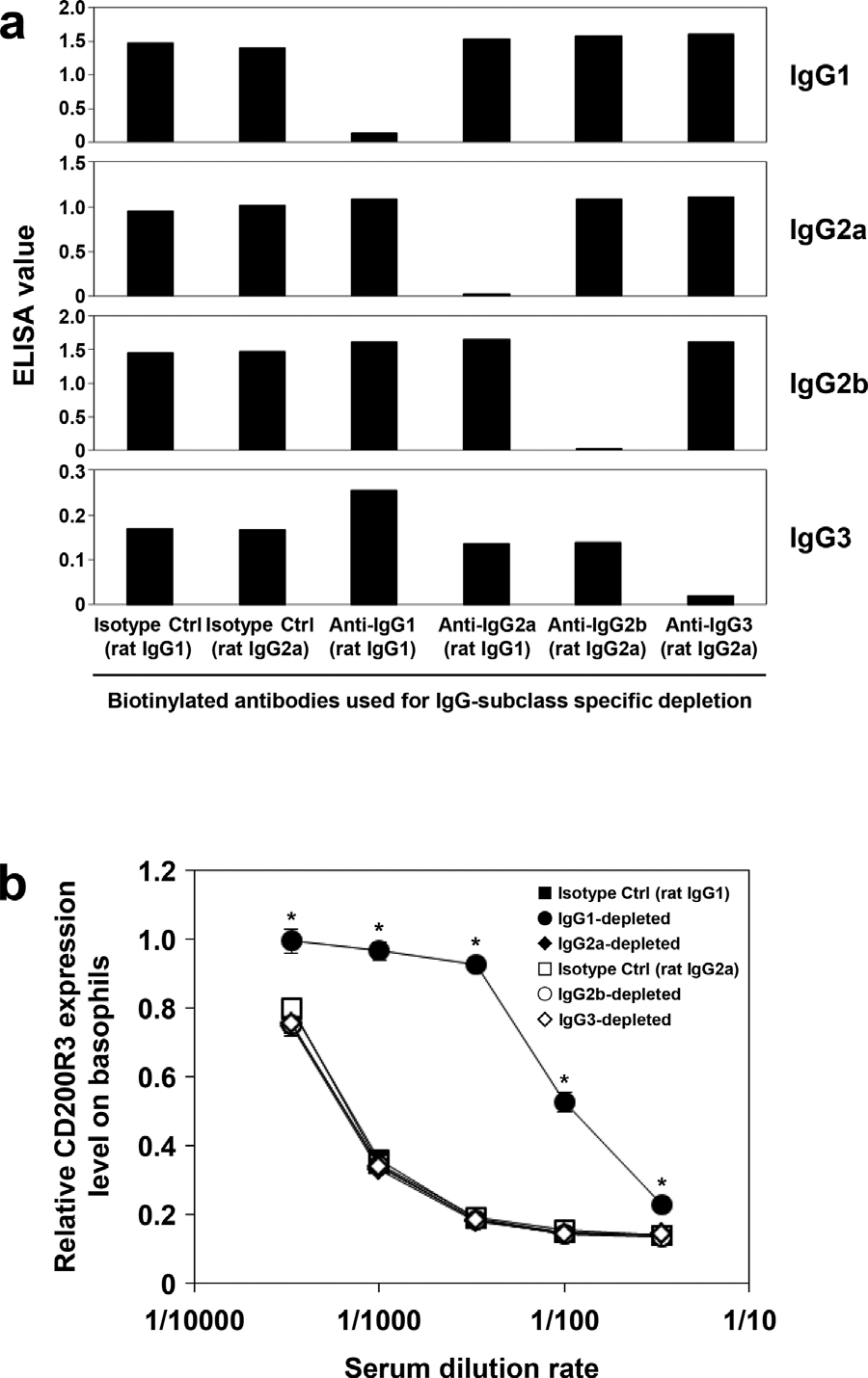
Basophil CD200R3 expression following depletion of specific IgG subclasses from antiserum used for passive sensitization. Each IgG subclass was depleted from mouse anti-β-LG serum using biotinylated antibodies specific to IgG1, IgG2a, IgG2b, IgG3, or corresponding isotype controls. (a) IgG subclass-specific depletion from antiserum was confirmed by ELISA. (b) Mouse peripheral blood was then sensitized with various dilutions of the depleted antisera. Antigen challenge followed by flow cytometry analysis was carried out as described in Figure 4. Significant differences compared to the isotype control group are indicated by asterisks (*P < 0.01).

To further determine the IgG-subclass influencing CD200R3 expression, anti-β-LG serum was subjected to IgG subclass-specific depletion and then used for passive sensitization. Subclass-specific depletion from antiserum was confirmed by ELISA (Fig. 5a). In terms of IgG1, its titer was decreased to a one-fifteenth by the treatment (data not shown). Only the depletion of IgG1 negated the down-regulation of CD200R3 strongly (Fig. 5b), the extent of this effect was proportional to the decrease in antibody titer. These results indicate that the decrease of CD200R3 on basophils is regulated by IgG1-specific mechanisms.

### IgG-mediated anaphylaxis is accompanied by decreased CD200R3 expression on basophils

**Figure 6:**
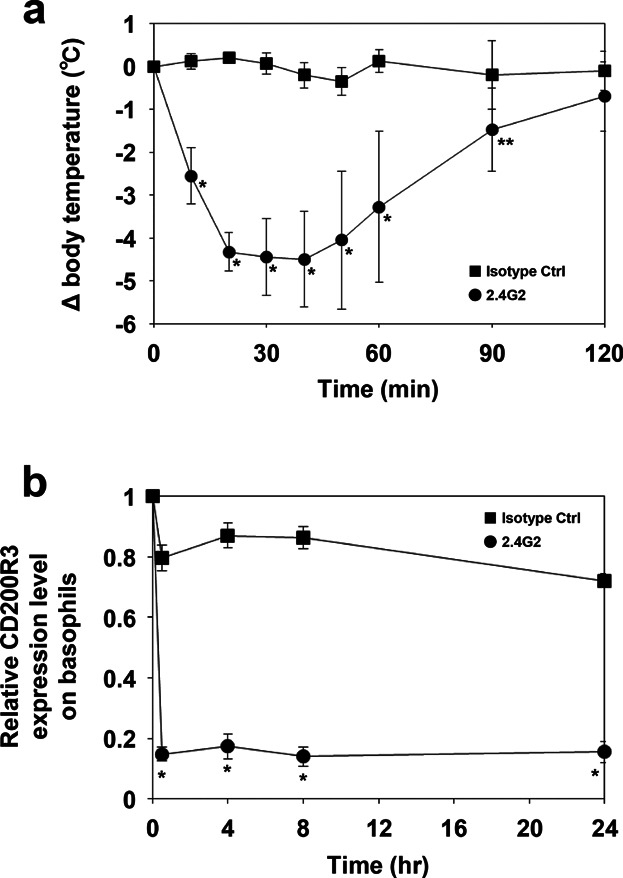
Decreased basophilic surface CD200R3 expression after induction of IgG-mediated anaphylaxis. (a) Mice were injected intravenously with 2.4G2 or isotype control, and rectal temperatures were monitored. Four mice per injection group were used. (b) The mice were then bled before injection (0 h), and 0.5, 4, 8, and 24 h afterwards. Basophil CD200R3 expression was subsequently evaluated. Data are represented as means ± SDs (*n* = 4). Asterisks indicate significant differences between the two groups at each time point (*P < 0.01, **P < 0.05).

It has been reported that the i.v. injection of anti-mouse FcγRIII/II mAb (2.4G2) induces systemic anaphylaxis in mice 7,8. To confirm that decreased CD200R3 expression on basophils constitutes a specific marker for IgG-mediated anaphylaxis, the change in CD200R3 levels in peripheral basophils after the induction of IgG receptor-mediated systemic anaphylaxis was evaluated. A significant decrease in rectal temperature was observed in mice injected with anti-FcγRIII/II mAb (Fig. 6a). A decrease in basophilic surface CD200R3 expression was detectable 30 min following antibody injection and continued for at least 24 h (Fig. 6b). These results indicate that down-regulation of CD200R3 on basophils could be used as an IgG-mediated anaphylaxis marker.

## Discussion

We observed not only increased expression of CD200R1 but also decreased expression of CD200R3 on the cell surface of sensitized basophils following stimulation by a specific antigen. As these changes were not always simultaneous, it was suggested that decreased expression of CD200R3 was not induced via an Fc∊RI-dependent process.

Anaphylaxis in mice can be brought about not only by an IgE-Fc∊RI mechanism but also through an IgG-FcγR interaction 3,4,7,29; therefore, we hypothesized that the decreased expression of CD200R3 was induced by FcγR-mediated stimulation. This hypothesis was strongly supported by the following findings: (i) CD200R3 on basophils sensitized with IgG-depleted antiserum was not down-regulated following specific antigen challenge (Fig. 4b), (ii) the level of CD200R3 on naive basophils was seen to decrease after stimulation with anti-FcγRIII/II mAb, (2.4G2; Fig. 3a), and (iii) the subclass-specific depletion experiment revealed that decreased expression of CD200R3 is dependent on IgG1 (Fig. 5b).

In the present study, we also found that anti-IgE antibody stimulation induced a minor decrease in CD200R3 expression on basophils (Fig. 2). It has been reported that mouse IgE has a certain level of affinity to both FcγRIIB and FcγRIII 30. A decrease in CD200R3 might be explained by IgE/anti-IgE complex-mediated IgG receptor cross-linking. Furthermore, stimulation with an anti-FcγRIII/II antibody (2.4G2) resulted in not only a pronounced decrease in CD200R3 expression, but also a statistically significant decrease in the level of CD200R1 on basophils. This down-regulation of CD200R1 could potentially be explained by the interaction between 2.4G2 and the inhibitory IgG Fc receptor, FcγRIIB.

There are many cell surface markers that can distinguish activated cells from resting ones. Although most of these are up-regulated upon cellular stimulation, some are down-regulated. The activation marker of human neutrophils, CD62L, is known to decrease after exposure to lipopolysaccharides and some bacterial lipoproteins 31,32. Our time-course study demonstrated that down-regulation of CD200R3 on basophils following FcγR-mediated stimulation occurs within 5 min and expression reaches its lowest level after 30–60 min (Fig. 3). The rapidly decreasing of CD200R3 expression is similar to that seen with CD62L on neutrophils, as mentioned above. In respect to murine cells expressing Fc receptors, FcγRIII on neutrophils and Bsp1 on basophils have been reported to be down-regulated by IgG- and IgE-mediated stimulation, respectively 11,33. However, the precise kinetics of their regulation remain uncertain. Prolonged stimulation, that is, for longer than 8 h, of mouse basophils in vitro with the β-LG immune-complex eventually led to a recovery of CD200R3 expression (data not shown). However, this recovery was not observed between 30 min and 24 h after i.v. injection of anti-FcγRIII/II mAb (Fig. 6b). The reason for this discrepancy between in vitro and in vivo time-course profiles remains to be investigated.

Basophils passively sensitized with mouse antiserum demonstrated down-regulation of CD200R3 following antigen challenge, and depletion of IgG1, but not IgG2a, IgG2b, or IgG3, from the antiserum negated any change in CD200R3 expression (Fig. 5b). This observation indicates that IgG1 is required for the down-regulation of CD200R3 on basophils. Among IgGs, IgG1, IgG2a, and IgG2b contribute to anaphylaxis by engaging FcγRs 10,34. IgG1 and IgG2 induce anaphylaxis via FcγRIII and FcγRIV, respectively 10. Although both are expressed on neutrophils, FcγRIII, but not FcγRIV, is also expressed on basophils and mast cells 10,30. Taken together, these reports and our findings suggest that the antigen/IgG1 immune complex activates basophils via FcγRIII and induces down-regulation of CD200R3 on the cell surface. In the present study, addition of an anti-FcγRIII/II mAb, 2.4G2, also reduced expression of CD200R3. As mouse basophils express FcγRIIB as well as FcγRIII, it remains to be clarified which of these two FcγRs plays the major role in down-regulation of CD200R3.

It is known that there are two major pathways in mouse anaphylaxis 4. One consists of IgE-stimulated histamine induction (the classical pathway), while the other comprises IgG-stimulation leading to platelet-activating factor (PAF) release (the alternative pathway). Tsujimura 5 reported that mouse basophils release PAF when stimulated by an antigen/IgG1 complex and that i.v. injection of PAF induces a decrease in rectal temperature in mice. We showed that in vivo stimulation of FcγRs induces systemic anaphylaxis (Fig. 6a) and down-regulation of CD200R3 on the surface of peripheral basophils (Fig. 6b). As part of the present investigation, we attempted to examine PAF release from CD49b^+^ splenocytes, crudely purified basophils. However, possibly due to the small number of basophils included in our study (we used splenocytes derived from three mice), PAF release could not be reliably determined. Although the contribution of basophils to IgG-mediated anaphylaxis in mice may be limited given their small numbers 10, it is probable that they release PAF 5 and reduce the presence of CD200R3 on their surfaces. This suggests that the down-regulation of CD200R3 on basophils could be used as a marker of IgG-mediated anaphylaxis in mice.

CD200R3 is a member of the CD200R family, present on mouse mast cells and basophils, and associated with the activating adaptor protein DAP12 28,35,36. It has been reported that stimulation via CD200R3 induces degranulation of mast cells, IL-4 production in basophils, and local and systemic anaphylaxis in mice 28. This implies that CD200R3 is functioning as an activating receptor. In the present study, an antigen challenge induced up-regulation of CD200R1 and down-regulation of CD200R3 on sensitized basophils. CD200R1 is known to be an inhibitory receptor 37–39; therefore, it has been speculated that its up-regulation functions as a negative-feedback mechanism in activated basophils 23. Although the biological implication of CD200R3 down-regulation on basophils stimulated via FcγRs is not clear, this receptor, together with CD200R1, may constitute a suppressive mechanism against excessive immune responses.

In conclusion, down-regulation of CD200R3 on basophils is induced by IgG-mediated stimulation via FcγRs. Use of CD200R3 as an activation marker, in addition to CD200R1, allowed us to evaluate murine basophil activation induced by IgG1 and IgE, respectively.

Human anaphylaxis is generally induced by IgE-mediated mast cell activation, but there is some evidence to suggest the existence of IgG-mediated anaphylaxis in humans 4,40–42. We expect that the establishment of a murine BAT system using CD200R3 will contribute to the advancement of research into IgG-induced anaphylaxis.
